# Comparative effectiveness of high-intensity interval training versus moderate-intensity continuous training in patients with type 2 diabetes mellitus: a systematic review and meta-analysis

**DOI:** 10.3389/fendo.2026.1833684

**Published:** 2026-05-13

**Authors:** Pengbo Ren, Jian Zhang, Shi Li, Tingwen Li

**Affiliations:** 1 Department of Physical Education, Changzhou University Huaide College, Jingiiang, China; 2 School of Physical Education, Minnan Normal University, Zhangzhou, China; 3 Sports Department, City College of Vocational Technology, Wuxi, China

**Keywords:** aerobic exercise, high-intensity interval training, meta-analysis, moderate-intensity continuous training, type 2 diabetes mellitus

## Abstract

**Objective:**

To systematically evaluate and compare the effects of High-Intensity Interval Training (HIIT) and Moderate-Intensity Continuous Training (MICT) on key glycemic indicators and related metabolic parameters in patients with Type 2 Diabetes Mellitus (T2DM).

**Methods:**

Databases including PubMed, Web of Science, Cochrane Library, CNKI, and Wanfang were searched to collect randomized controlled trials (RCTs) published up to October 2025 on HIIT and MICT in T2DM. Two researchers independently screened the literature, extracted data, and assessed the risk of bias in the included studies. A meta-analysis was then performed using RevMan 5.4.

**Results:**

A total of 21 RCTs involving 831 T2DM patients were included. The meta-analysis results showed that compared to a normal control group, both HIIT and MICT significantly reduced fasting blood glucose (FBG) and increased patients’ VO_2_max levels. HIIT significantly reduced patients’ glycated hemoglobin (HbA_1_c) levels, body mass index (BMI), and significantly increased high-density lipoprotein (HDL) levels. Compared to MICT, HIIT was more effective in reducing FBG and provided a greater increase in maximal oxygen uptake (VO_2_max). Both HIIT and MICT effectively reduced HbA_1_c in T2DM patients, although there was no significant difference in the effect between the two modalities. Regarding BMI, low-density lipoprotein (LDL), and HDL, the two exercise modalities did not show statistically significant differences.

**Conclusion:**

In the management of T2DM, HIIT offers greater advantages in improving FBG and enhancing VO_2_max, providing a basis for the scientific and effective management of T2DM. However, due to the limitations in the number and quality of the included studies, the above conclusions require verification through more high-quality research.

## 1 Introduction

Type 2 Diabetes Mellitus (T2DM) has evolved into a chronic global pandemic, with its prevalence increasing rapidly and posing a severe public health challenge. According to the latest data from the International Diabetes Federation (IDF), approximately 537 million adults worldwide have diabetes, of which T2DM accounts for over 90%. This number is projected to rise to 783 million by 2045 (1). Notably, the disease is trending towards affecting younger populations and is growing fastest in low- and middle-income countries. China, a major epicenter of T2DM, has a staggering 141 million patients, ranking highest globally. The prevalence rate has reached 11.2%, yet awareness and control rates remain comparatively low. This large-scale epidemic imposes a heavy disease burden. T2DM and its complications are leading causes of cardiovascular disease, kidney failure, blindness, and non-traumatic lower-limb amputations, placing immense pressure on healthcare systems and socio-economies. Curbing the spread of T2DM has become a global health priority (2). The core pathophysiological features of T2DM include insulin resistance and relative insulin deficiency, leading to persistent hyperglycemia. This, in turn, triggers various serious complications such as cardiovascular disease, nephropathy, retinopathy, and neuropathy (2). Therefore, seeking effective, safe, and sustainable blood glucose management strategies is fundamental to the comprehensive treatment of T2DM.

In T2DM management, lifestyle intervention is considered a first-line strategy equally important as pharmacotherapy. Among these interventions, regular physical exercise is widely proven to improve insulin sensitivity, promote glucose uptake, control body weight, and optimize body fat distribution, serving as an independent protective factor for glycemic control (3). For a long time, Moderate-Intensity Continuous Training (MICT), also known as aerobic exercise (AE) such as brisk walking, jogging, and cycling, has been recommended as the standard exercise prescription for T2DM patients in most clinical guidelines due to its high safety profile and ease of implementation (4). In recent years, High-Intensity Interval Training (HIIT), as a more time-efficient exercise modality, has garnered widespread attention in academia and clinical practice. HIIT typically consists of short bursts of vigorous activity interspersed with periods of low-intensity recovery or rest. Existing research suggests that HIIT may offer unique advantages in improving cardiorespiratory fitness, mitochondrial biogenesis, and insulin sensitivity (5). Some preliminary randomized controlled trials (RCTs) indicate that compared to traditional MICT, HIIT may provide equivalent or even superior glycemic control benefits for T2DM patients, particularly in reducing glycated hemoglobin (HbA_1_c) and postprandial blood glucose (6, 7).

Although a considerable number of original studies have explored the efficacy of HIIT versus MICT for T2DM, their conclusions remain inconsistent. Some studies suggest HIIT is significantly superior to MICT in improving parameters like fasting blood glucose (FBG) (8), while others indicate that the two modalities yield comparable benefits in reducing body fat percentage and fasting glucose levels, with no statistically significant difference (9). This inconsistency may stem from heterogeneity across studies in terms of population characteristics, intervention duration, specific HIIT protocols (e.g., work/rest ratios, intensity), and control group settings. Therefore, in the context of this ongoing debate in the evidence, it is particularly necessary to employ systematic review and meta-analysis methods to quantitatively synthesize and comprehensively evaluate existing RCTs. Previous meta-analyses have compared the effects of HIIT and MICT on cardiovascular function, body mass index (BMI), VO_2_max, etc., in T2DM patients. However, systematic analyses incorporating multiple indicators such as blood glucose, blood lipids, and exercise capacity are scarce. Furthermore, existing comparisons are often limited to HIIT vs. MICT, lacking simultaneous comparison with a non-exercise control group (10, 11). Consequently, this study aims to systematically evaluate the effects of HIIT and MICT on key glycemic indicators (e.g., HbA_1_c, FBG), maximal oxygen uptake (VO_2_max), low-density lipoprotein (LDL), and BMI in T2DM patients through rigorous literature search and quality assessment. It also seeks to compare the relative effectiveness of the two exercise modalities. The findings of this study are expected to provide higher-level evidence-based medical evidence for formulating individualized exercise prescriptions for T2DM patients and offer a reference for updating clinical guidelines.

## 2 Data and methods

### 2.1 Inclusion and exclusion criteria

The inclusion and exclusion criteria were established based on the PICOS framework (**Table 1**) (CRD: 420251271763).

**Table 1:** Inclusion and exclusion criteria.

PICOS elements	Inclusion criteria	Exclusion criteria
P(Population)	**1. Population:** Adult patients (≥18 years) with a confirmed diagnosis of T2DM. **2. Diagnostic Criteria:** Consistent with internationally recognized diagnostic criteria for T2DM (e.g., American Diabetes Association, WHO standards).	1. Patients with type 1 diabetes, gestational diabetes mellitus, or other specific types of diabetes. 2. Patients with severe diabetic complications (e.g., end−stage renal disease, active retinopathy causing visual impairment, etc.). 3. Patients with other severe systemic diseases (e.g., heart failure, severe respiratory diseases, cancer) or musculoskeletal disorders that preclude participation in exercise interventions.
**I**(Intervention)	Both of the following interventions are implemented concurrently, with a total intervention duration of no less than 12 weeks. The following training sessions are performed daily: **1. HIIT** A structured exercise protocol consisting of alternating short periods of high−intensity exercise with active/passive recovery phases, with each session lasting 30–40 minutes. **2. MICT** Continuous AE performed at moderate intensity, such as brisk walking, jogging, cycling, etc., with each session lasting 30–60 minutes.	1. Non-structured exercise protocols or protocols without clearly defined intervals. 2. Interventions that incorporate other specific therapies (e.g., specialized dietary therapy, physiotherapy) that may significantly influence outcomes, with discrepancies between the experimental and control groups.
**C**(Comparison)	**The study must include one of the following control conditions:** **1. Non-exercise control group** Participants receive only standard advice regarding exercise guidelines for T2DM and do not engage in any organized physical activity. **2. MICT** Continuous AE performed at moderate intensity, such as brisk walking, jogging, cycling, etc.	1. Other types of exercise as controls (e.g., resistance training, flexibility training). 2. Single-arm studies without a control group.
**O**(Outcome)	Primary outcomes: HbA_1_c (%), FBG (mmol/L) Secondary outcomes: VO_2_max (L/min), LDL (mmol/L), HDL (mmol/L), BMI (kg/m²)	The outcome indicators of the research report do not include any relevant blood glucose or metabolic indicators.
**S**(Study Design)	**RCTs**	Non-randomized controlled studies, observational studies (cohort studies, case-control studies), reviews, case reports, conference abstracts, unpublished dissertations, and studies for which full texts are unavailable.

HIIT, High-intensity interval training; MICT, Moderate-intensity continuous training; RCTs, Randomized controlled trials.

### 2.2 HIIT and MICT exercise programs

HIIT is implemented using two methods: long intervals and short intervals. In the long-interval approach, the exercise phase lasts three to five minutes, followed by a 3-5-minute recovery phase. This cycle is repeated four to six times, resulting in a total duration of 30–40 minutes (including warm-up and cool-down). For the short-interval approach, the exercise phase lasts 0.5 to one minute, followed by a one to two minutes recovery phase, repeated eight to twelve times, with a total duration of approximately 20–30 minutes (including warm-up and cool-down). HIIT sessions are conducted one to three times per week. During the exercise phase, the target heart rate should reach 80-95% of maximum heart rate or 70-90% of maximum oxygen uptake. During the recovery phase, the heart rate should decrease to 40-60% of the maximum heart rate (9, 12, 13). MICT includes AE such as brisk walking, jogging, cycling, and swimming, is a classic form of exercise intervention. It requires maintaining a heart rate at 50-70% of the maximum heart rate or a perceived exertion level described as slightly breathless but able to converse. Each session lasts 30–60 minutes and is performed three to five times per week (9, 12, 13). The control group receives only standard counseling on exercise guidelines for T2DM and does not participate in any organized physical activity.

### 2.3 Literature search strategy

Computerized searches of the PubMed, Web of Science, Cochrane Library, China National Knowledge Infrastructure (CNKI), and Wanfang databases were conducted, with the search deadline set as October 2025. Additionally, the reference lists of included studies were reviewed manually. The search terms included: “type 2 diabetes mellitus,” “aerobic exercise,” “Moderate-Intensity Continuous Training,” “high-intensity interval training,” and “randomized controlled trial” (**Supplementary Material 1**).

### 2.4 Literature screening and data extraction

Literature screening and data extraction were independently conducted by two researchers, AA and BB, followed by cross-verification to ensure accuracy. In cases of disagreement, the researchers initially attempted to resolve discrepancies through in-depth discussion to reach consensus. If consensus could not be achieved, a third researcher, CC, was consulted to ensure an objective and impartial resolution. The extracted information included: first author’s name, publication year, country, proportion of males, average age, total number of cases, interventions, outcome measures.

### 2.5 Quality assessment

To systematically evaluate the risk of bias in the included literature, this study employed RoB 2.0 recommended by the Cochrane Collaboration. The assessment comprehensively and rigorously examined the following domains: selection bias (randomization process), reporting bias (selective outcome reporting), performance bias (blinding of participants and personnel), detection bias (blinding of outcome assessment), attrition bias (incomplete outcome data), and other potential sources of bias. This approach ensured a thorough and rigorous evaluation process.

### 2.6 Statistical methods

The meta-analysis in this study was conducted by using RevMan 5.4. Additionally, funnel plots were generated to assess potential publication bias. For continuous variables, the standardized mean difference (SMD) and its 95% confidence interval (CI) were used as effect size measures. The *I*² test was employed to evaluate statistical heterogeneity among studies, which was classified into three levels: high (*I*² > 50%), moderate (25% < *I*² ≤ 50%), and low (*I*² ≤ 25%). If moderate or high heterogeneity was observed, the DerSimonian-Laird random-effects model was applied; otherwise, the Mantel-Haenszel fixed-effects model was used. Publication bias was assessed using Begg’s funnel plot, and sensitivity analysis was performed using the leave-one-out method in STATA software.

## 3 Results

### 3.1 Literature search results

**Figure 1:**
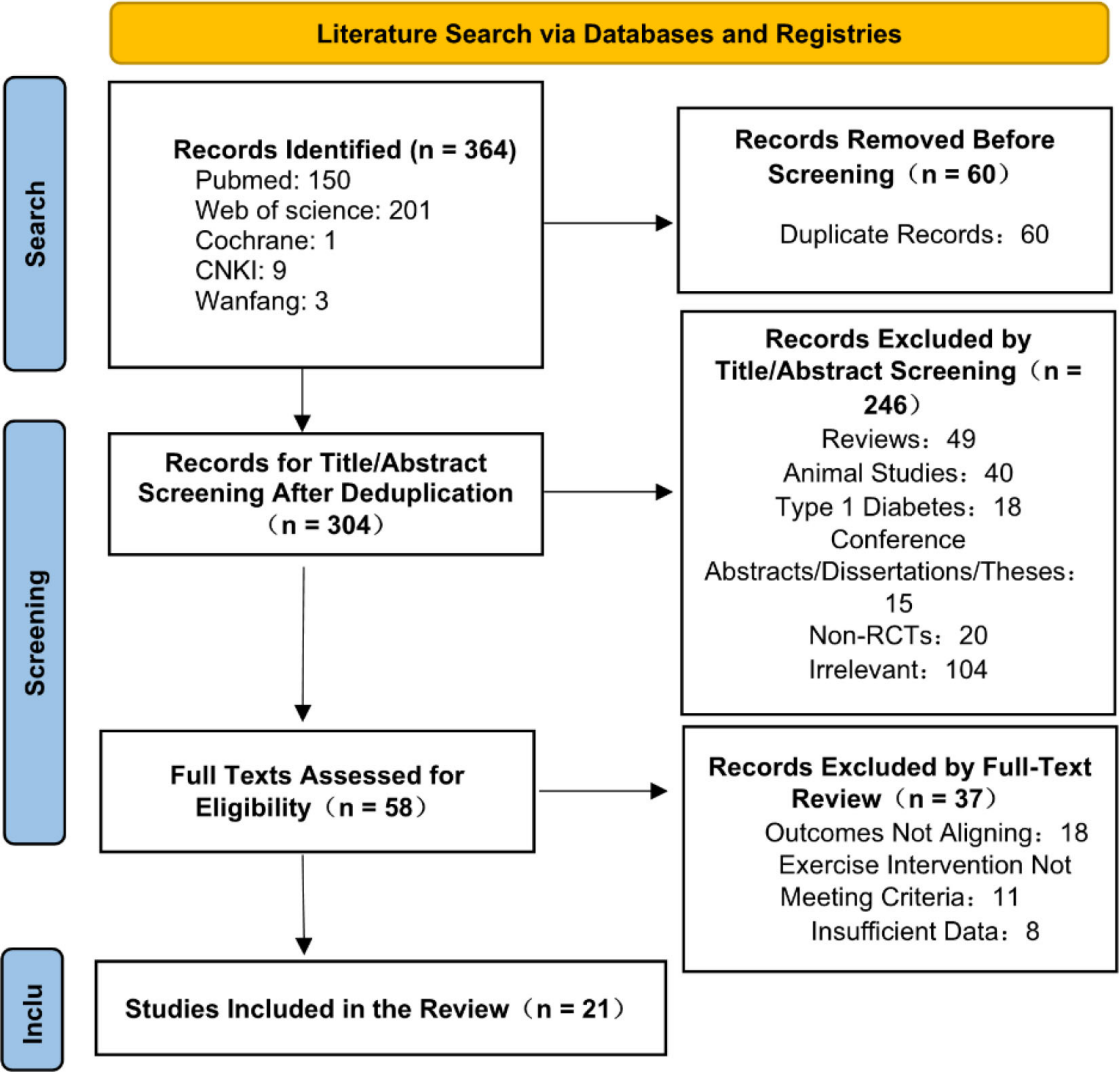
PRISMA flowchart of literature screening.

A total of 364 records were identified through the literature search for this study. After removing duplicates, 304 articles remained. Subsequently, an initial screening of titles and abstracts was conducted based on predetermined exclusion criteria, which led to the exclusion of 246 articles, including reviews, animal studies, those focused on type 1 diabetes, conference abstracts, and non-randomized controlled trials. The full texts of the remaining 58 articles were independently reviewed by two researchers, resulting in the exclusion of an additional 37 articles. The primary reasons for exclusion were outcomes not aligning with the study’s main objectives, exercise intervention methods not meeting inclusion criteria, and insufficient available data. Ultimately, 21 RCTs were included for qualitative and quantitative synthesis. The literature screening process is illustrated in **Figure 1** and summarized in **Table 2**.

**Table 2:** Basic characteristics of the included studies.

Included studies	Exercise type	Intervention frequency	Sample size	Male proportion(%)	Age	Outcome indicators
Li J, 2021 (12)	HIIT/MICT/control	5 days a week (15 min per session for HIIT; 30 min per session for MICT)	13/12/12	100/100/100	38.0 ± 6.0 39.0 ± 6.0 40.0 ± 7.0	FBG/HbA_1_c/BMI/VO_2_max
Mitranum W,2014 (9)	MICT/HIIT/control	3 days a week(30–40 min per session)	14/14/15	35.7/35.7/33.3	61.7 ± 2.7 61.2 ± 2.8 60.9 ± 204	FBG/HbA_1_c/BMI/VO_2_max/LDL/HDL
Su C, 2024 (13)	HIIT/MICT/control	Every day (≥30 min per session)	76/77/75	48.0/48.7/50.6	61.4 ± 5.2 61.7 ± 6.2 61.2 ± 6.0	HbA_1_c/BMI
Magalhaes JP,2018 (14)	HIIT/MICT/control	3 days a week(33–45 min per session)	25/28/27	60.0/46.4/51.9	56.7 ± 8.3 59.7 ± 6.5 59.0 ± 8.1	FBG/HbA_1_c/BMI/VO_2_max
Banitalebi E,2019 (15)	HIIT/MICT/control	3 days a week(50 min per session)	14/14/14	0.0/0.0/0.0	55.4 ± 5.9 54.1 ± 5.4 55.7 ± 6.4	FBG/HbA_1_c/BMI/VO_2_max
Baasch-Skytte T,2019 (16)	HIIT/MICT	3 days a week(29 min per session for 10-20–30 training; 50 min per session for MICT)	23/21	100/100	61.0 ± 6.2 61.2 ± 7.1	FBG/HbA_1_c/BMI/VO_2_max
Teles GO, 2022 (17)	HIIT/MICT	2 times a week (18–24 min per session);	14/15	NR	54.6 ± 8.9 57.3 ± 8.9	FBG
Strijcker DD,2018 (18)	HIIT/MICT	2 times a week(40 min per session)	8/8	100/100	47.0 ± 3.4 46.0 ± 5.6	FBG/BMI/VO_2_max
Wormgoor SG, 2018 (19)	HIIT/MICT	3 days a week (60 min per session)	12/11	100/100	52.2 ± 7.1 52.5 ± 7.0	HbA_1_c/BMI/VO_2_max
Mendes R, 2019 (20)	HIIT/MICT/control	1 session per condition (40 min per session)	15/15/15	46.7/46.7/46.7	60.2 ± 3.1 60.5 ± 3.4 60.1 ± 3.2	FBG
Stoa EM, 2017 (21)	HAIT/MICT	3 days a week (52–60 min per session)	19/19	60.5/60.5	59.0 ± 10.0 59.0 ± 11.0	HbA_1_c/BMI/VO_2_max/LDL/HDL
Martins FM,2018 (22)	HIIT/MICT	3 days a week (28–60 min per session)	8/8	0.0/0.0	64.3 ± 6.7 65.0 ± 6.3	FBG/HbA_1_c
Maillar F, 2016 (8)	HIIT/MICT	2 days a week (20–40 min per session)	8/9	0.0/0.0	/	FBG/HbA_1_c/BMI/LDL/HDL
Ryckeghem LV, 2022 (23)	HIIT/MICT	3 days a week (duration not specified)	10/9	90.0/88.9	61.0 ± 5.0 66.0 ± 11.0	FBG/HbA_1_c/BMI/VO_2_max
Chen K, 2024 (24)	HIIT/MICT/control	Daily (5 days per week, 2 rest days after every 5 consecutive days) (10–30 min per session)	12/10/11	25.0/24.4/27.3	63.7 ± 8.6 62.2 ± 8.0 59.7 ± 12.2	FBG/LDL/HDL
Rafiei H, 2019 (25)	HIIT/MICT	1 session per condition (25–30 min per session)	8/7	0.0/0.0	45.18 ± 8.5 46.0 ± 12.9	FBG/BMI/VO_2_max
Robinson E, 2015 (26)	HIIT/MICT	5 days a week (20–50 min per session)	20/18	25.0/21.0	52.0 ± 10.0 52.0 ± 10.0	FBG
Dunnwald T, 2018 (27)	HIIT/MICT	3 days a week (42–50 min per session)	8/6	75.0/66.7	59.6 ± 5.7 59.5 ± 6.0	FBG/BMI/VO_2_max
Liu X, 2020 (28)	HIIT/MICT	3 days a week (20–50 min per session)	72/73	45.8/46.6	68.2 ± 7.6 69.8 ± 6.3	FBG/BMI/VO_2_max/LDL/HDL
Winding KM, 2018 (6)	HIIT/MICT/control	3 days a week (20–40 min per session)	13/12/7	71.4/58.3/53.8	54.0 ± 6.0 58.0 ± 8.0 57.0 ± 7.0	FBG/HbA_1_c/BMI/VO_2_max LDL
Costa-Arruda RM, 2025 (29)	HIIT/MICT	1 session per condition (24 min per session)	30/27	NR	NR	FBG

FBG, Fasting Blood Glucose; HIIT, High-Intensity Interval Training; MICT, Moderate-Intensity Continuous Training; control, control group without organized exercise; HbA_1_c, Glycated Hemoglobin; BMI, Body Mass Index; VO_2_max, Maximal Oxygen Uptake; LDL, Low-Density Lipoprotein; HDL, High-Density Lipoprotein; NR, Not Reported.

### 3.2 Characteristics of the included studies

A total of 21 studies were included in this review, comprising 460 participants in the HIIT group, 340 participants in the MICT group, and 128 participants in the control group. The basic characteristics of these studies are presented in **Table 1**. The age of the sample ranged from 38.0 to 69.8 years. Four studies included only male participants (12, 16, 18, 19), while another four studies included only female participants (8, 15, 22, 25). In the remaining studies, the proportion of male participants ranged from 21.0% to 90.0%. No significant differences were observed in age or gender distribution between the groups. Among the included studies, eight compared the effects of the three types of exercise (HIIT, MICT, and control) on the outcome indicators (6, 9, 12–15, 20, 24), while 13 studies directly compared HIIT and MICT (8, 16–19, 21–23, 25–29). The duration of both HIIT and MICT interventions was at least 12 weeks. In the HIIT group, one session of HIIT (lasting 30–40 minutes) was performed daily, whereas in the MICT group, one session of MICT (lasting 30–60 minutes) was performed daily.

### 3.3 Assessment of methodological quality of included literature

**Figure 2:**
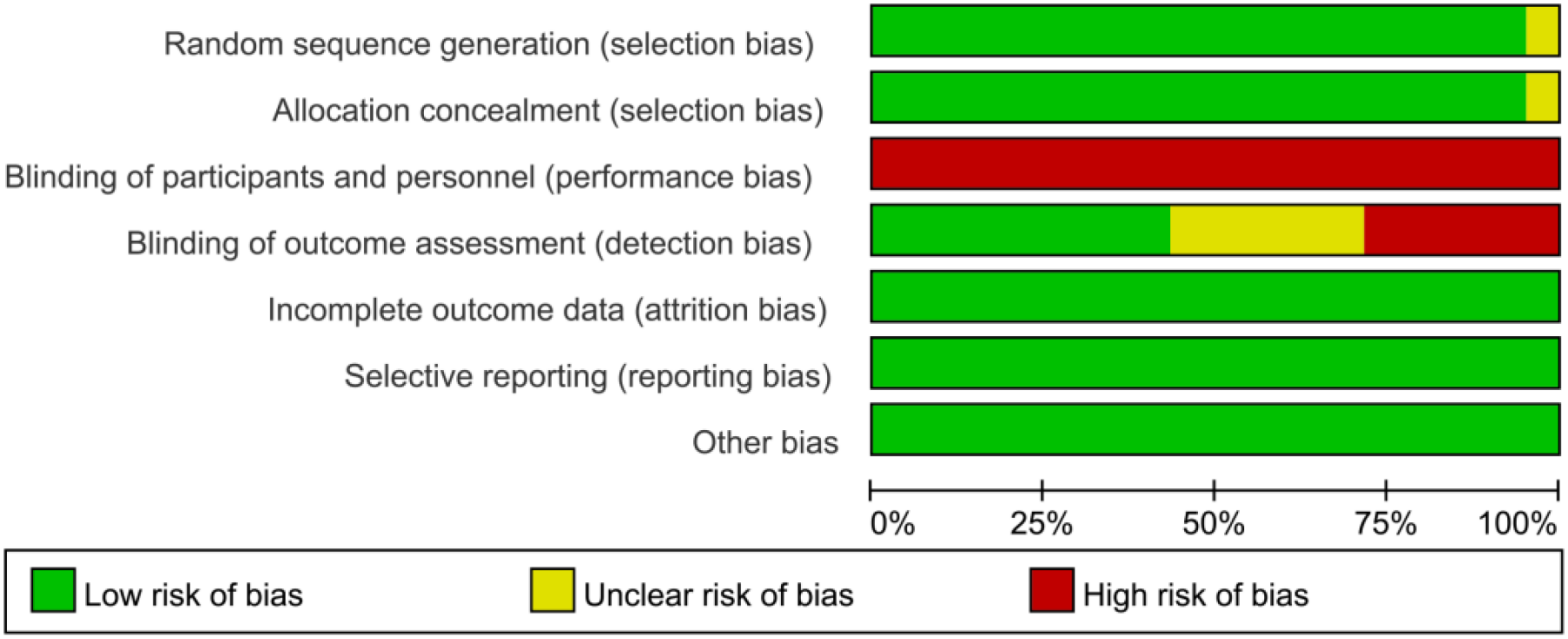
Risk of bias graph.

**Figure 3:**
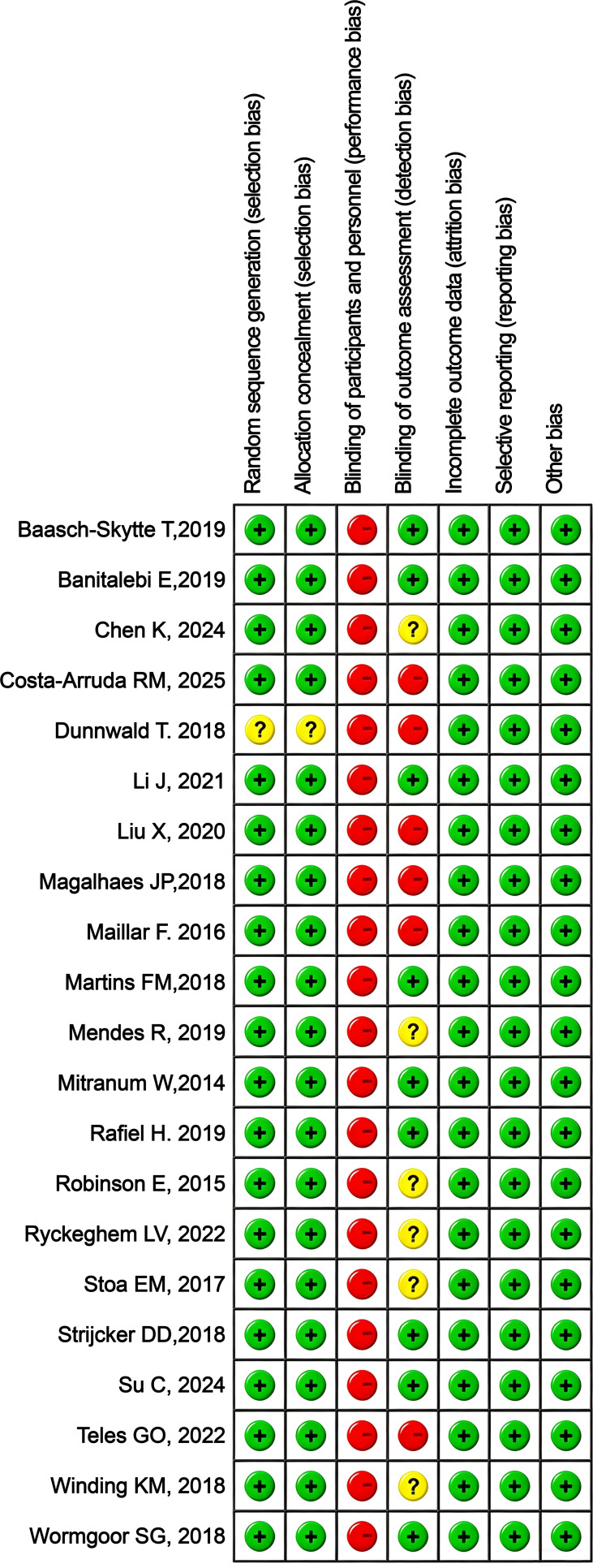
Summary graph of risk of bias for each study.

All 21 RCTs included in this systematic review reported the baseline characteristics of the participants in detail. Furthermore, each study elaborated on the specific randomization process, intervention protocols, and outcome assessment measures, while also providing a detailed description of the grouping methods. However, due to the specific nature of exercise intervention studies, none of the studies implemented blinding during the intervention process. For laboratory or sample testing, most studies either applied blinding or did not mention it. The results of the risk of bias analysis are presented in **Figures 2**, **3**.

### 3.4 Meta-analysis results: HIIT vs. MICT

#### 3.4.1 Effect of HIIT vs. MICT on FBG

**Figure 4:**
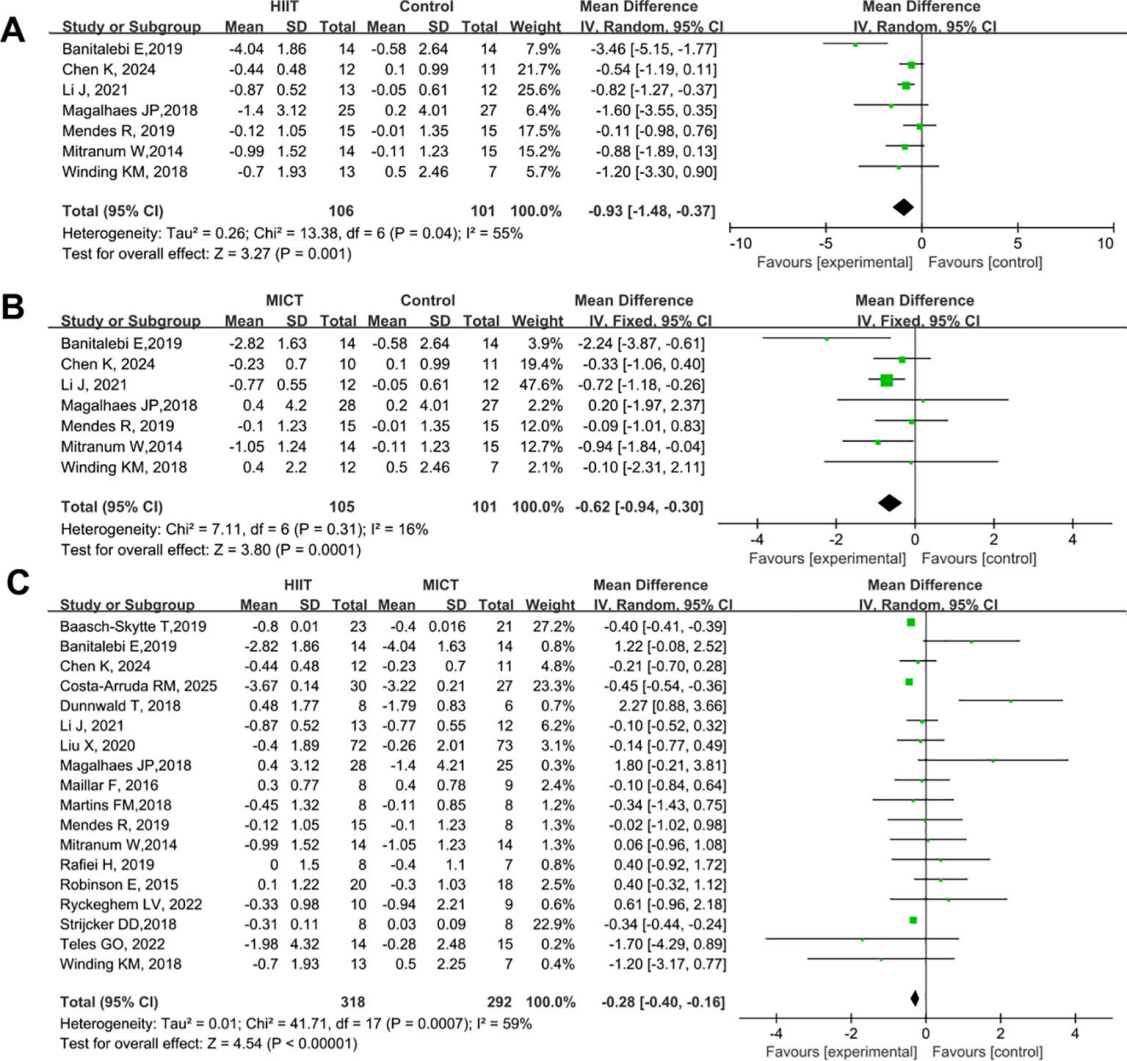
Forest plot analysis of the effects of different exercise modes on FBG changes in patients with T2DM. **(A)** HIIT versus non-exercise control group; **(B)** MICT versus non-exercise control group; **(C)** HIIT versus MICT group. FBG, Fasting Blood Glucose; T2DM, Type 2 Diabetes Mellitus; HIIT, High-Intensity Interval Training; MICT, Moderate-Intensity Continuous Training.

A total of 18 studies were included to compare the effects of HIIT and MICT on FBG in patients with T2DM. The results are shown in **Figure 4**. Compared with the control group, both HIIT and MICT significantly reduced FBG (HIIT vs. control: SMD = -0.93, 95% CI: -1.48 to -0.37, P = 0.001; MICT vs. control: SMD = -0.62, 95% CI: -0.94 to -0.30, P = 0.0001) (**Figures 4A, B**). The blood glucose-lowering effect of HIIT was significantly superior to that of MICT (HIIT vs. MICT: SMD = -0.28, 95% CI: -0.40 to -0.16, P < 0.00001) (**Figure 4C**).

#### 3.4.2 Effect of HIIT vs. MICT on HbA_1_c

**Figure 5:**
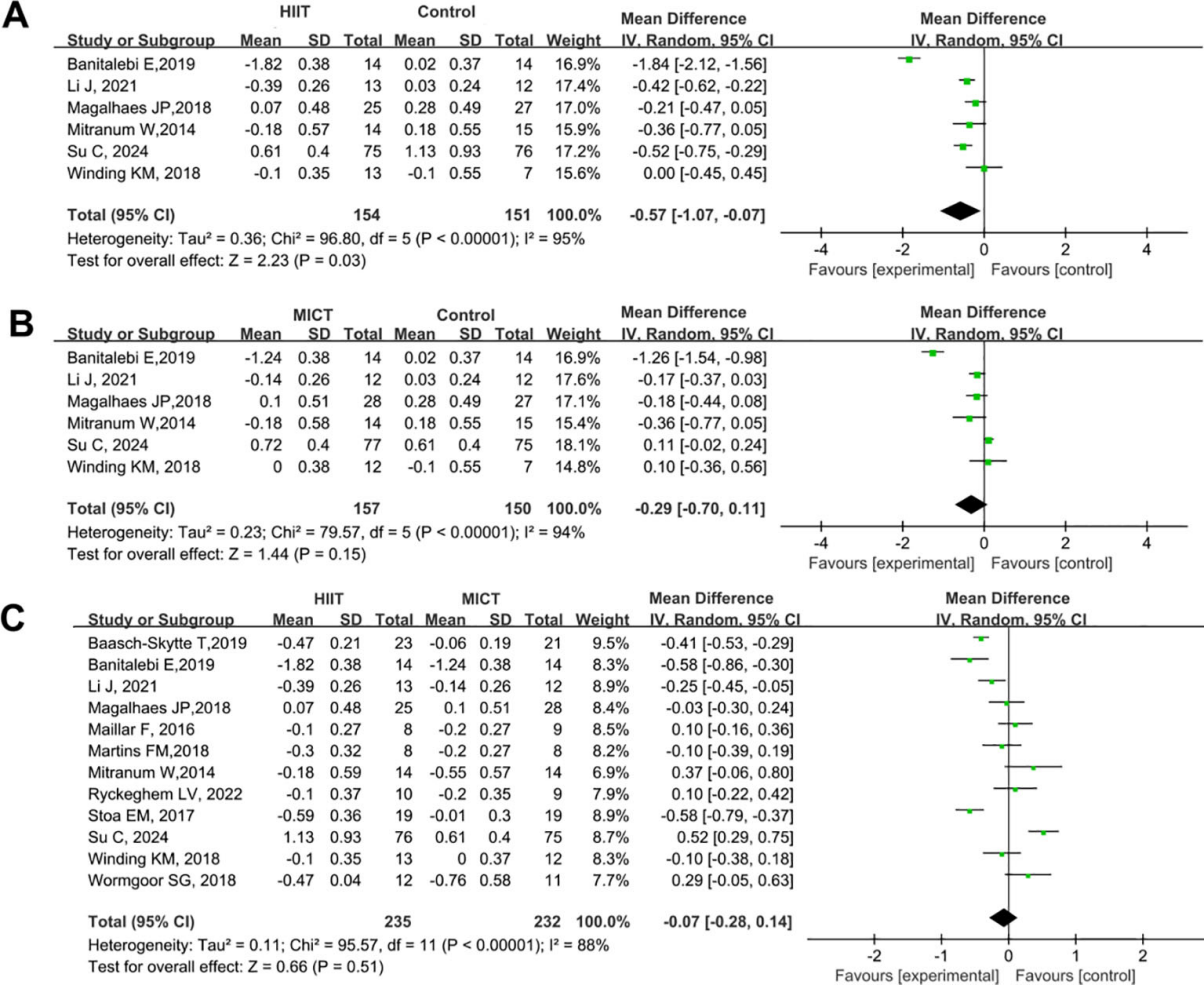
Forest plot analysis of the effects of different exercise modes on HbA1c changes in patients with T2DM. **(A)** HIIT versus non-exercise control group; **(B)** MICT versus non-exercise control group; **(C)** HIIT versus MICT group. HbA1c, Glycated Hemoglobin; T2DM, Type 2 Diabetes Mellitus; HIIT, High-Intensity Interval Training; MICT, Moderate-Intensity Continuous Training.

A total of 12 studies were included to compare the effects of HIIT and MICT on HbA_1_c in patients with T2DM. The results are presented in **Figure 5**. Compared with the control group, HIIT significantly reduced HbA_1_c levels (SMD = -0.57, 95% CI: -1.07 to 0.07, P = 0.03) (**Figure 5A**). In contrast, MICT did not show a significant reduction in HbA_1_c levels (SMD = -0.29, 95% CI: -0.70 to 0.11, P = 0.15) (**Figure 5B**). No significant difference was observed between the HIIT and MICT groups in terms of their effect on HbA_1_c (HIIT vs. MICT: SMD = -0.07, 95% CI: -0.28 to 0.14, P = 0.51) (**Figure 5C**).

#### 3.4.3 Effect of HIIT vs. MICT on BMI

**Figure 6:**
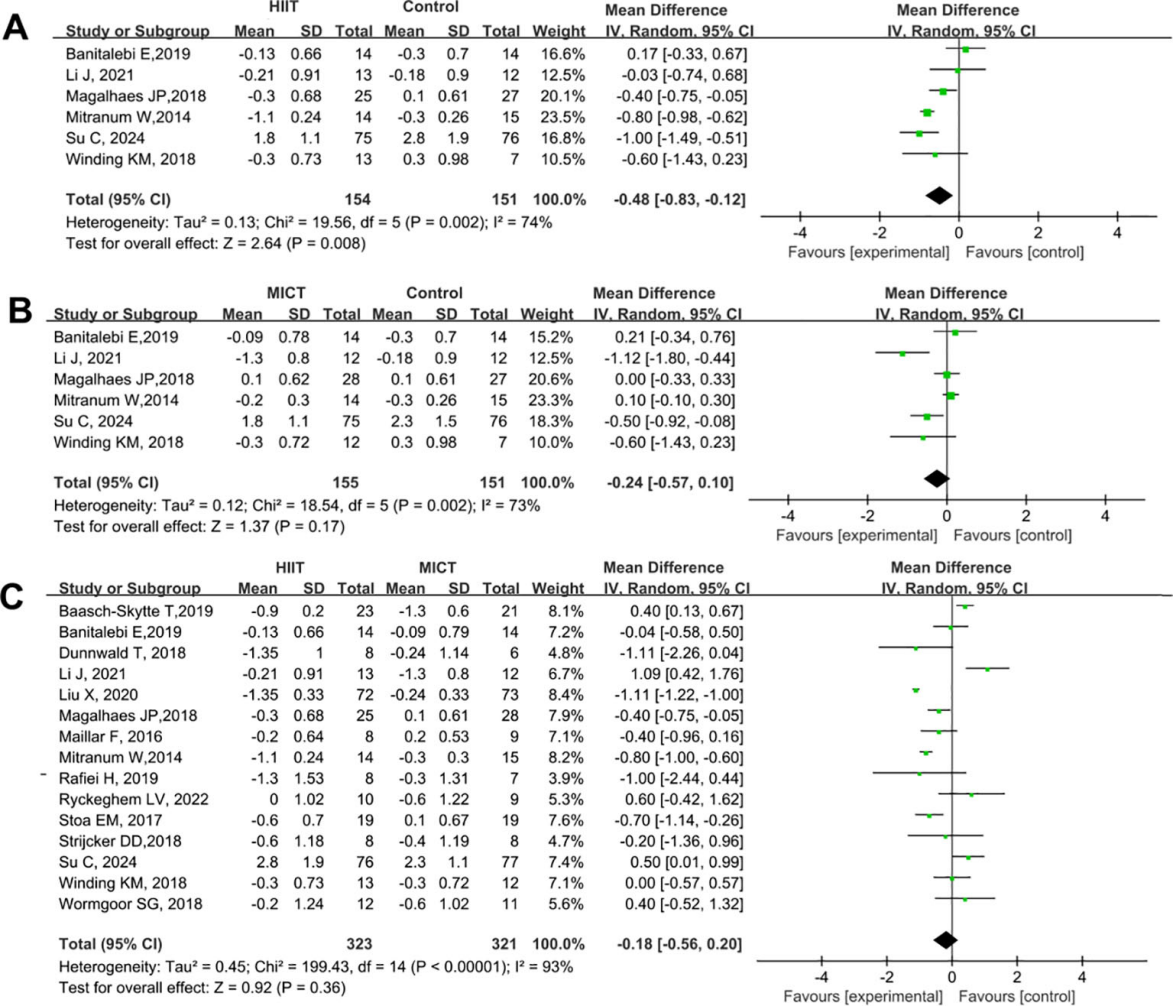
Forest plot analysis of the effects of different exercise modes on BMI changes in patients with T2DM. **(A)** HIIT versus non-exercise control group; **(B)** MICT versus non-exercise control group; **(C)** HIIT versus MICT group. BMI, Body Mass Index; T2DM, Type 2 Diabetes Mellitus; HIIT, High-Intensity Interval Training; MICT, Moderate-Intensity Continuous Training.

A total of 15 studies were included to compare the effects of HIIT and MICT on BMI in patients with T2DM. The results are shown in **Figure 6**. Compared with the control group, HIIT significantly reduced BMI (SMD = -0.48, 95% CI: -0.83 to -0.12, P = 0.008) (**Figure 6A**), whereas MICT did not have a significant effect on BMI (SMD = -0.24, 95% CI: -0.57 to 0.10, P = 0.17) (**Figure 6B**). No significant difference was observed between the HIIT and MICT groups in their effects on BMI (HIIT vs. MICT: SMD = -0.18, 95% CI: -0.37 to 0.20, P = 0.36) (**Figure 6C**).

#### 3.4.4 Effect of HIIT vs. MICT on LDL

**Figure 7:**
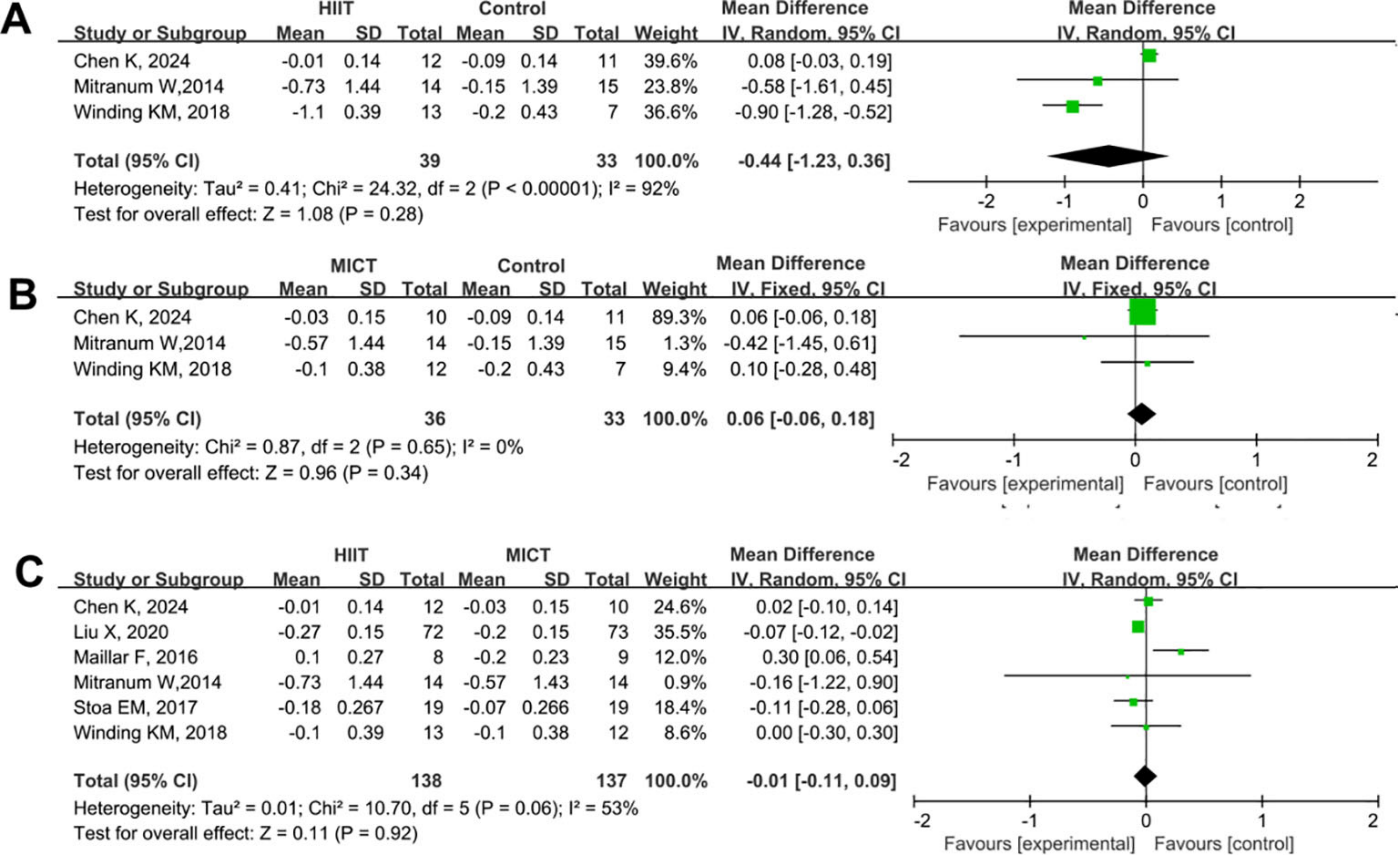
Forest plot analysis of the effects of different exercise modes on low-density lipoprotein (LDL) changes in patients with T2DM. **(A)** HIIT versus non-exercise control group; **(B)** MICT versus non-exercise control group; **(C)** HIIT versus MICT group. LDL, Low-Density Lipoprotein; T2DM, Type 2 Diabetes Mellitus; HIIT, High-Intensity Interval Training; MICT, Moderate-Intensity Continuous Training.

A total of six studies were included to compare the effects of HIIT and MICT on LDL in patients with T2DM. The results are shown in **Figure 7**. Compared with the control group, neither HIIT nor MICT showed a significant effect on LDL (HIIT vs. control: SMD = -0.44, 95% CI: -1.23 to 0.36, P = 0.28; MICT vs. control: SMD = 0.06, 95% CI: -0.06 to 0.18, P = 0.34) (**Figures 7A, B**). No significant difference was observed between the HIIT and MICT groups in their effects on LDL (HIIT vs. MICT: SMD = -0.01, 95% CI: -0.11 to 0.09, P = 0.92) (**Figure 7C**).

#### 3.4.5 Effect of HIIT vs. MICT on HDL

**Figure 8:**
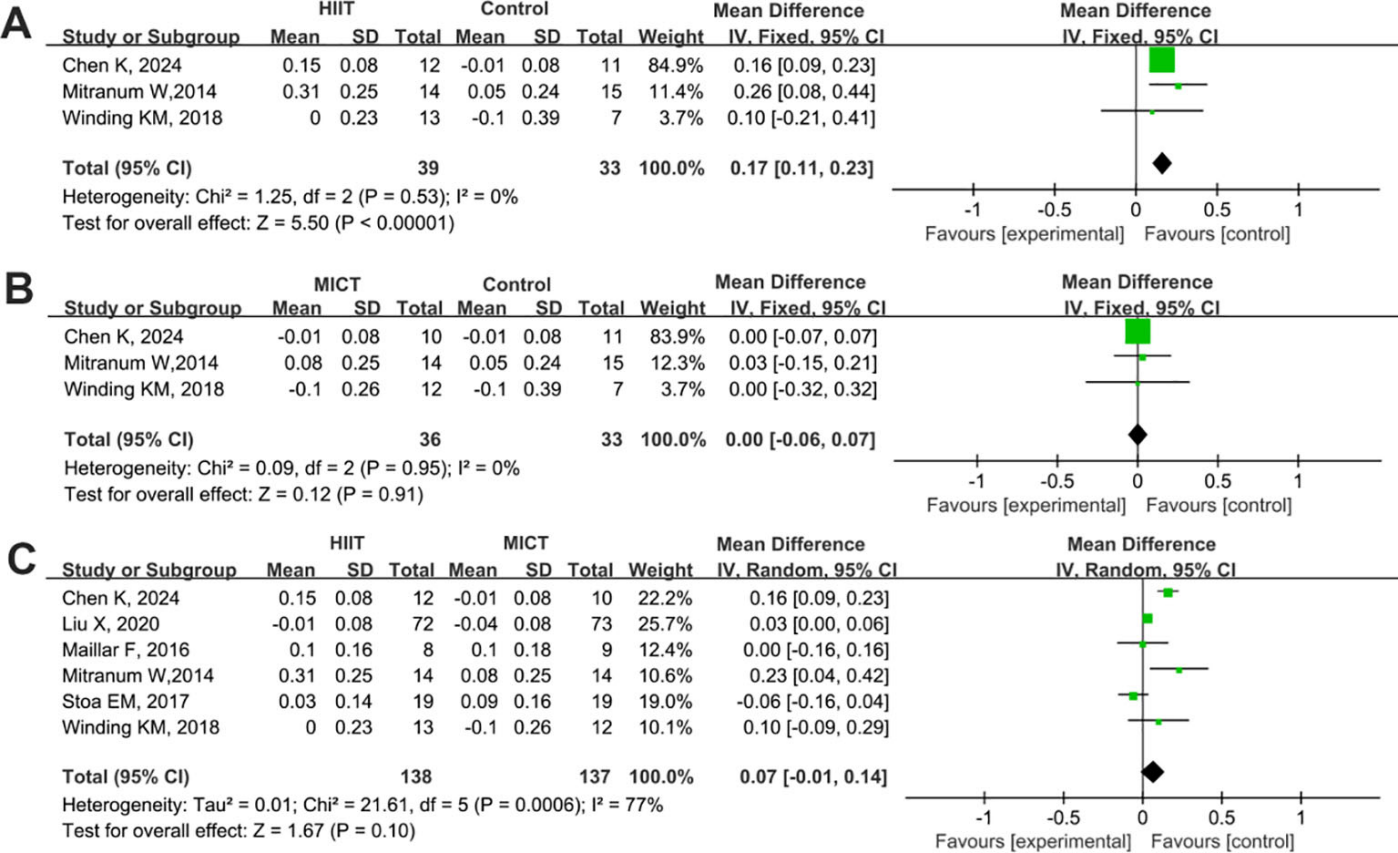
Forest plot analysis of the effects of different exercise modes on high-density lipoprotein (HDL) changes in patients with T2DM. **(A)** HIIT versus non-exercise control group; **(B)** MICT versus non-exercise control group; **(C)** HIIT versus MICT group. HDL, High-Density Lipoprotein; T2DM, Type 2 Diabetes Mellitus; HIIT, High-Intensity Interval Training; MICT, Moderate-Intensity Continuous Training.

A total of six studies were included to compare the effects of HIIT and MICT on HDL in patients with T2DM. The results are shown in **Figure 8**. Compared with the control group, HIIT significantly increased HDL levels (HIIT vs. control: SMD = 0.17, 95% CI: 0.11 to 0.23, P < 0.00001) (**Figure 8A**), while MICT showed no significant effect on HDL (MICT vs. control: SMD = 0.00, 95% CI: –0.06 to 0.07, P = 0.91) (**Figure 8B**). No significant difference was observed between the HIIT and MICT groups in their effects on HDL (HIIT vs. MICT: SMD = 0.07, 95% CI: –0.01 to 0.14, P = 0.10) (**Figure 8C**).

#### 3.4.6 Effect of HIIT vs. MICT on VO_2_max

**Figure 9:**
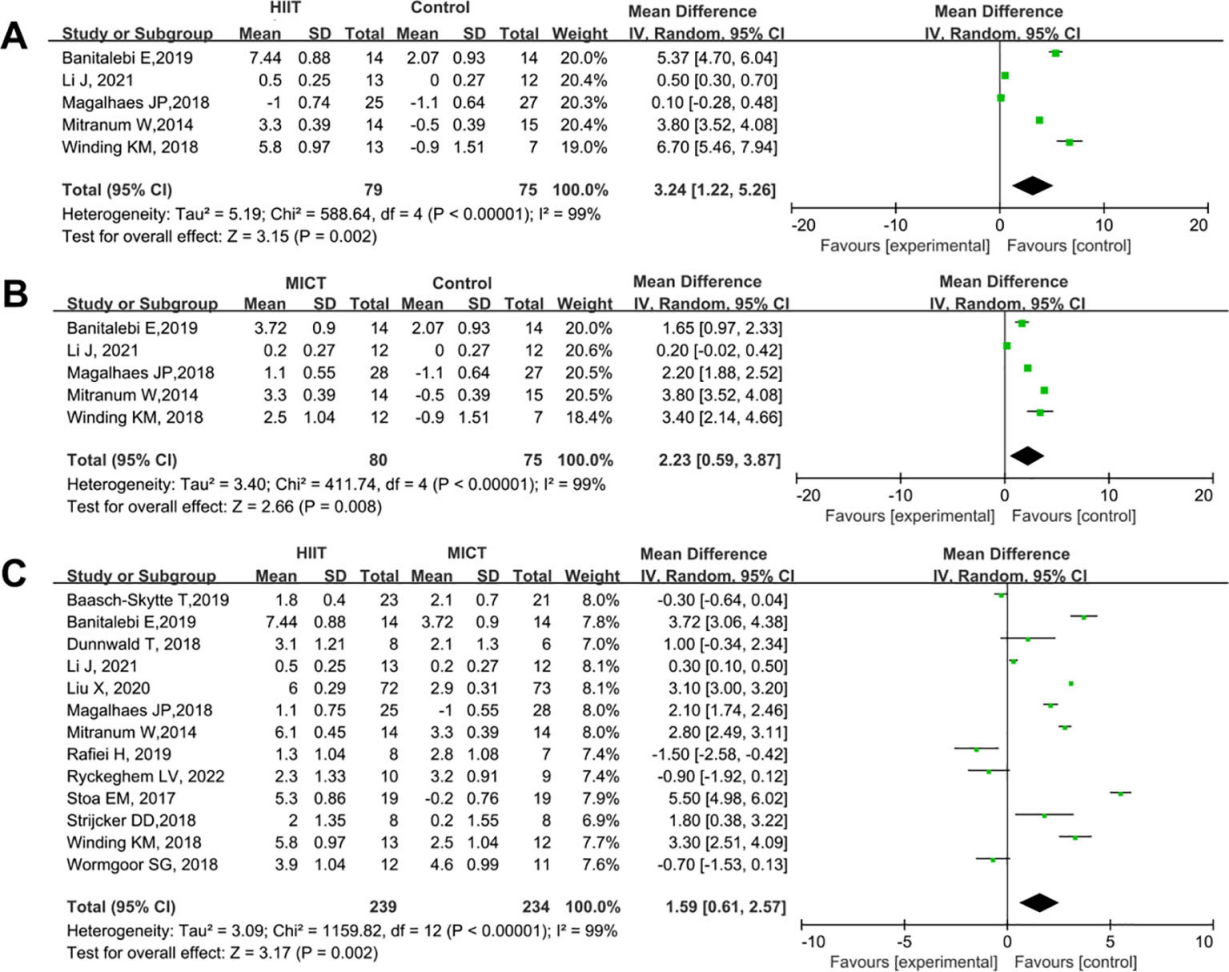
Forest plot analysis of the effects of different exercise modes on maximal oxygen uptake (VO2max) changes in patients with T2DM. **(A)** HIIT versus non-exercise control group; **(B)** MICT versus non-exercise control group; **(C)** HIIT versus MICT group. VO₂max, Maximal Oxygen Uptake; T2DM, Type 2 Diabetes Mellitus; HIIT, High-Intensity Interval Training; MICT, Moderate-Intensity Continuous Training.

A total of 13 studies were included to compare the effects of HIIT and MICT on VO_2_max in patients with T2DM. The results are shown in **Figure 9**. Compared with the control group, both HIIT and MICT significantly increased VO_2_max levels (HIIT vs. control: SMD = 3.24, 95% CI: 1.22 to 5.26, P = 0.002; MICT vs. control: SMD = 2.23, 95% CI: 0.59 to 3.87, P = 0.008) (**Figures 9A, B**). The improvement in VO_2_max was significantly greater in the HIIT group than in the MICT group (HIIT vs. MICT: SMD = 1.59, 95% CI: 0.61 to 2.57, P = 0.002) (**Figure 9C**).

### 3.5 Publication bias

**Figure 10:**
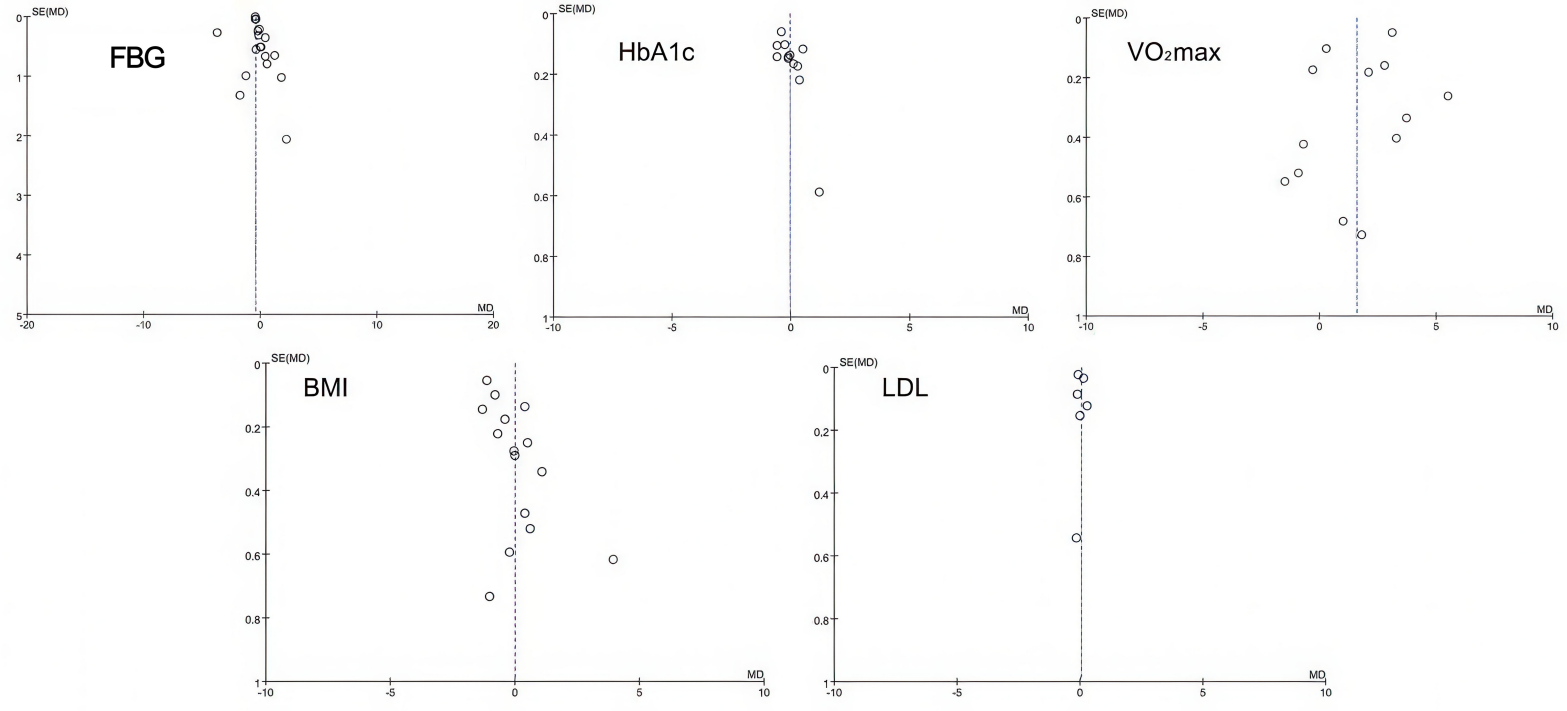
Publication bias funnel plot.

Publication bias was assessed using funnel plots. The results indicated that the distribution of study points showed no significant asymmetry, suggesting a low likelihood of substantial publication bias. The corresponding results are presented in **Figure 10**.

### 3.6 Sensitivity analysis

**Figure 11:**
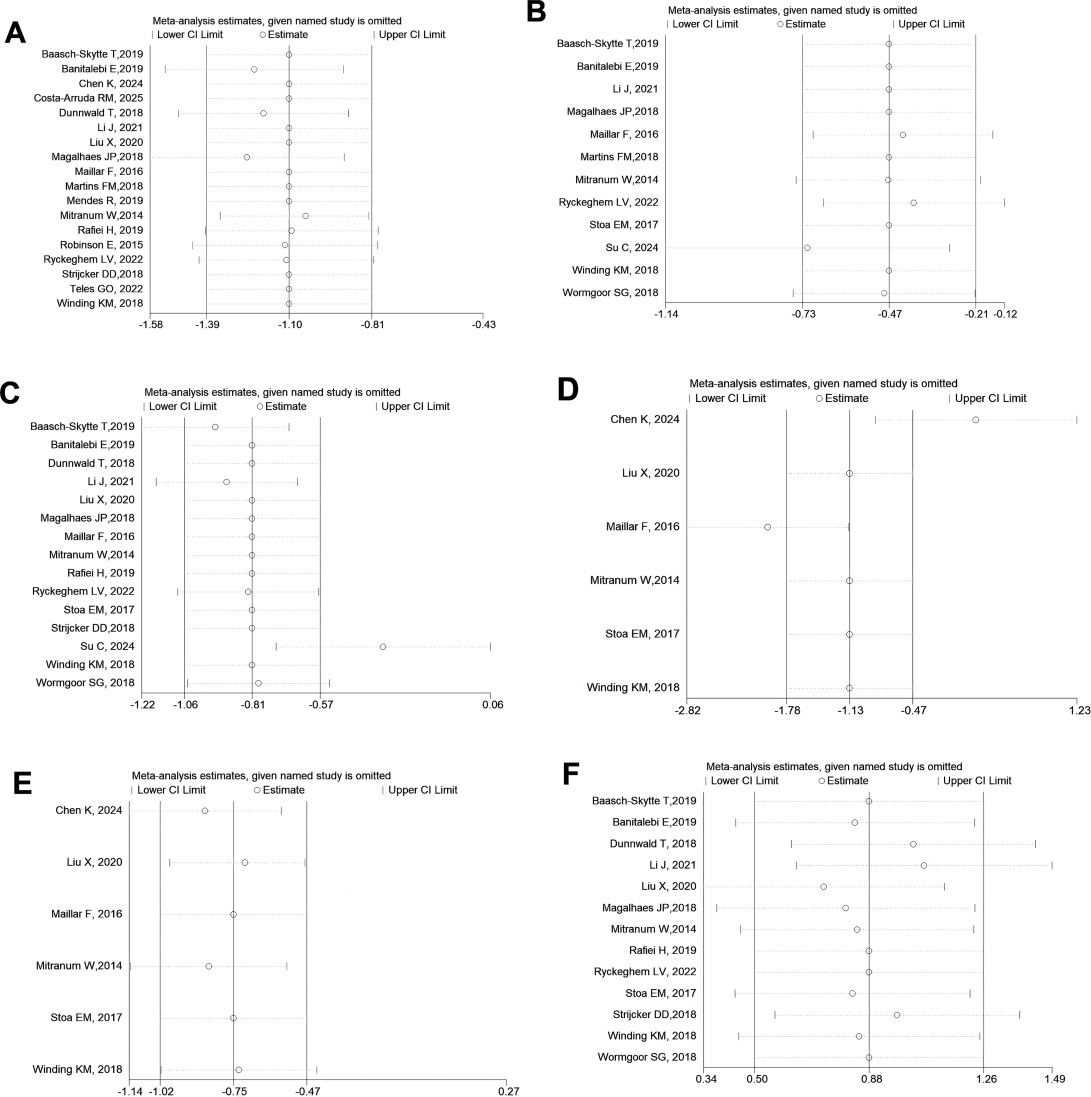
Sensitivity analysis of the comparison between HIIT and MICT. **(A)** Fasting Blood Glucose (FBG); **(B)** Glycated Hemoglobin (HbA1c); **(C)** Body Mass Index (BMI); **(D)** Low-Density Lipoprotein (LDL); **(E)** High-Density Lipoprotein (HDL); **(F)** Maximal Oxygen Uptake (VO_2_max).

Sensitivity analysis was performed using the leave-one-out method in STATA. The results, as shown in **Figure 11**, indicate that in the HIIT vs. control group, the removal of Su C (13) changed the effect on BMI from statistically significant (before removal) to non-significant, though heterogeneity remained unaffected (**Figure 11C**). Meanwhile, the exclusion of studies by Chen K (24) and Maillar F (8) led to a significant reduction in heterogeneity for LDL (**Figure 11D**). For FBG, HbA_1_c, HDL, and VO_2_max, the removal of any individual study did not alter the overall results (**Figures 11A, B**, **E, F**).

## 4 Discussion

This study systematically evaluated and synthesized, through a systematic review and meta-analysis, the effects of HIIT and MICT on FBG, HbA_1_c, BMI, LDL, HDL, and VO_2_max in patients with T2DM. The findings indicate that, compared with the control group, both HIIT and MICT significantly reduced FBG and increased VO_2_max levels. HIIT notably lowered HbA_1_c levels, reduced BMI, and significantly improved HDL levels. Compared with MICT, HIIT was more effective in reducing FBG and produced a greater improvement in VO_2_max. Both HIIT and MICT effectively reduced HbA_1_c in patients with T2DM; however, no significant difference was observed between the two interventions in terms of HbA_1_c reduction. Regarding BMI, LDL, and HDL, no statistically significant differences were found between the two exercise modalities. These results suggest that HIIT holds greater advantages in improving FBG and enhancing cardiorespiratory fitness, thereby providing evidence for the scientific and effective management of T2DM.

This meta-analysis found that both HIIT and MICT can significantly reduce FBG, and HIIT is significantly superior to MICT in lowering FBG. FBG primarily reflects hepatic insulin sensitivity and the regulation of glucose output in the basal state. Compared to the relatively moderate MICT, the intense bursts of HIIT instantaneously consume a large amount of energy, rapidly depleting glycogen stores in the muscles. Post-exercise, muscle cells become extremely sensitive to insulin and continuously absorb glucose from the bloodstream, converting it into glycogen for storage, leading to a more pronounced reduction in FBG (30). The significant improvement in FBG observed in this study confirms that HIIT is superior to MICT in reducing FBG.

This meta-analysis found that HIIT can significantly lower HbA_1_c in patients with T2DM. While MICT can also reduce HbA_1_c levels, the difference was not statistically significant. When directly comparing HIIT and MICT, there was no statistically significant difference between them in lowering HbA_1_c. Due to its strong physiological stimulus, HIIT can significantly improve insulin sensitivity and glycemic control in a short period, often leading to a significant decrease in HbA_1_c when compared solely to baseline. Although MICT also has beneficial effects, when analyzed alone, the effect size may be smaller, potentially due to limited sample sizes or high individual variability, resulting in a statistically non-significant difference. HbA_1_c reflects mean glycemic levels over the preceding two to three months. Studies have demonstrated that when total energy expenditure is equated, the incremental benefits of higher exercise intensity on HbA_1_c reduction may be attenuated. This suggests that the amelioration of HbA_1_c levels may be primarily contingent upon the long-term accumulation of exercise (represented by cumulative energy expenditure), rather than transient exercise intensity per se (19). The advantage of HIIT may be its time efficiency; the total exercise time required for HIIT to achieve similar health benefits is typically much shorter than that for MICT. For example, while MICT requires 150 minutes of exercise per week, HIIT may only need 75 minutes to achieve comparable effects.

This meta-analysis pooled data from 15 studies and found that compared to MICT, HIIT can significantly reduce BMI levels in patients with T2DM, although we did not further identify a statistically significant difference in BMI improvement between the two interventions. Elevated BMI, particularly reaching overweight or obese criteria (e.g., BMI > 24 kg/m²), is one of the strongest independent risk factors for T2DM. Excess body fat, especially visceral fat, releases large amounts of free fatty acids and inflammatory factors, interfering with insulin signaling in tissues like the liver and muscles, leading to severe insulin resistance (12). Studies indicate that both HIIT and MICT can reduce BMI in T2DM patients, but their characteristics and efficiency differ (12). HIIT employs a “work-rest-work” cycle pattern (e.g., 30-second all-out sprint followed by 1-minute rest). Its advantage lies not only in energy expenditure during exercise but also inducing a significant post-exercise excess oxygen consumption (EPOC) effect, potentially leading to higher total energy expenditure (TEE) and theoretically being more conducive to fat loss (31, 32). Some studies suggest HIIT may be more effective in reducing visceral fat storage, thereby indirectly affecting BMI (31). High-intensity exercise stimulates the sympathetic nervous system, promotes catecholamine secretion, and enhances lipolysis (33). Therefore, HIIT is superior to MICT in terms of BMI reduction effectiveness.

This meta-analysis included 6 studies evaluating the effects of HIIT and MICT on LDL and HDL in T2DM patients. The results found that HIIT can significantly increase HDL levels but has no significant effect on LDL levels. MICT showed no significant effect on either HDL or LDL levels. Furthermore, there was no significant difference between HIIT and MICT in altering LDL and HDL levels. An increase in HDL is considered beneficial for T2DM patients. HDL exerts cardiovascular protective effects in T2DM patients through its antioxidant capacity and cholesterol efflux function. However, the mechanism by which HIIT significantly increases HDL levels has not been specifically studied. Interestingly, several studies have shown that HIIT also significantly reduces LDL levels in T2DM patients (6, 9). Alterations in lipid profiles generally necessitate more prolonged intervention durations (exceeding 12 weeks) and a substantial total volume of exercise (34). In cases where the study period is relatively short and dietary intake is not strictly controlled, marginal improvements in blood lipids may be obscured by inter-individual variability. The upregulation of LPL activity is a fundamental mechanism underlying the improvement of lipid profiles. Although HIIT induces more vigorous muscle contractions, MICT facilitates a higher cumulative stimulus for LPL through extended exercise durations. This discrepancy in metabolic stimulus potentially elucidates why both modalities often demonstrate comparable efficacy in improving lipid profiles (35, 36). The results of the study did not show a significant result, possibly due to the limited number of studies and small sample sizes (only three studies included, with 39 participants in the HIIT group and 33 in the control group). Sensitivity analysis revealed that removing the study by Chen K (24) significantly reduced heterogeneity, and the result changed from non-significant to significant, suggesting that the heterogeneity of individual studies might also have contributed to the instability of the pooled LDL result.

This meta-analysis also found that both HIIT and MICT can significantly improve patients’ VO_2_max levels, and HIIT is also clearly superior to MICT in enhancing VO_2_max (SMD = 1.59). VO_2_max is the gold standard for assessing cardiorespiratory fitness and the body’s oxygen utilization capacity. Its improvement is closely associated with a reduced risk of cardiovascular disease and all-cause mortality. Both HIIT and MICT can improve VO_2_max by enhancing cardiopulmonary oxygen delivery, peripheral oxygen utilization, and blood oxygen-carrying capacity. However, the high-intensity phases of HIIT (e.g., 80%-100% of maximum heart rate) directly push the body close to or to its VO_2_max level, providing an “extreme stimulus” to the cardiorespiratory system and muscle tissues. Following high-intensity exercise, it triggers EPOC, where the body consumes substantial oxygen to replenish ATP and phosphocreatine reserves, clear lactate, and restore body temperature and hormone levels. With long-term, repeated HIIT, this repeated “breakdown-rebuild” cycle forces the body to develop more efficient enzyme systems and mitochondrial networks, thereby permanently increasing VO_2_max (32, 37). Therefore, HIIT is ultimately significantly superior to MICT in rapidly improving VO_2_max.

This study has several limitations: First, there was considerable heterogeneity in the protocols. Sensitivity analysis using the one-study-removal method found that except for LDL, this heterogeneity could not be eliminated, indicating it was not caused by any single study. The included HIIT protocols varied greatly in work/rest ratios, sprint duration per bout, total number of cycles, etc., which may have increased heterogeneity in the pooled effect sizes (e.g., high I²values), affecting the ability to provide precise prescription recommendations for clinical practice. Second, the trials were not blinded. Due to the nature of exercise interventions, blinding participants and intervention providers (coaches, researchers) is extremely difficult, potentially introducing placebo/expectation effects among participants or providers, which may affect result reliability. Third, the sample sizes were limited. Among the 21 included studies, most had small sample sizes (n < 20 per group), which may have increased the risk of Type II error, potentially failing to detect low but clinically meaningful differences in some outcomes (e.g., the effects of HIIT vs. MICT on HbA_1_c, BMI, LDL).

Furthermore, the clinical trials included in this analysis may not have adequately controlled several critical confounding factors, such as the smoking status and genetic background. Smoking represents a major preventable risk factor significantly associated with increased all-cause and cause-specific mortality (38). It may also attenuate the potential benefits of exercise interventions by impairing cardiorespiratory and metabolic functions (38). Notably, the relationship between smoking and disease risk appears to be complex and potentially bidirectional. For instance, emerging genetic evidence suggests a paradoxical correlation between smoking and a reduced risk of certain neurological disorders, underscoring the necessity for rigorous control of smoking status in clinical studies (39). Additionally, there is substantial inter-individual heterogeneity in the physiological response to exercise training, in which genetic factors likely play a pivotal role. Evidence indicates that specific genetic polymorphisms (e.g., ACE and ACTN3) are closely associated with aerobic endurance performance, while genome-wide association studies have identified specific genetic variants linked to both athletic performance and injury susceptibility (40, 41). These findings suggest that genetic background may modulate physiological adaptations, the magnitude of therapeutic benefits, and even the risk of injury during HIIT or MICT.

## 5 Conclusion

This meta-analysis confirms that in the management of T2DM, HIIT holds greater advantages in improving FBG and enhancing VO_2_max, providing a basis for the scientific and effective management of T2DM. Due to limitations in the quantity and quality of the included studies, the above conclusions need to be verified by more high-quality research.
